# A population analysis of self‐management and health‐related quality of life for chronic musculoskeletal conditions

**DOI:** 10.1111/hex.12422

**Published:** 2015-11-02

**Authors:** Elizabeth A. Hoon, Tiffany K. Gill, Clarabelle Pham, Jodi Gray, Justin Beilby

**Affiliations:** ^1^ School of Public HealthThe University of AdelaideAdelaideSAAustralia; ^2^ School of MedicineThe University of AdelaideAdelaideSAAustralia; ^3^ Torrens University AustraliaAdelaideSAAustralia

**Keywords:** health‐related quality of life, musculoskeletal conditions, population health, self‐management

## Abstract

**Background:**

There is growing policy emphasis on self‐management as an essential component of musculoskeletal chronic care models. Underpinning this drive is the assumption that with correct ‘informational’ framing people will better manage their condition's progression and thereby maintain quality of life.

**Objective:**

To assess associations between self‐management behaviours and health‐related quality of life for people with chronic musculoskeletal conditions.

**Design:**

Using survey data from health census and follow‐up structured telephone interviews, linear regression (cumulatively adjusted for potential confounders) and logistic regression examined associations between use of specific self‐management behaviours and quality of life.

**Setting and participants:**

A total of 885 respondents (2012) who indicated still having a musculoskeletal condition reported in a 2010 health census (Port Lincoln, South Australia).

**Variables:**

Specific self‐management activities, age, sex, education, marital status, smoking, comorbidities and pain.

**Outcome measure:**

EQ‐5D‐5L.

**Results:**

Exercise (63%) and diet (19%) were the most commonly reported self‐management activities used to manage musculoskeletal conditions. About 24% reported not using any specific self‐management activities. Involvement in self‐management showed no association with quality of life, with and without adjustment for confounders. Diet had a negative association with quality of life as did use of formal support (self‐management course or community group support).

**Discussion:**

Taking a real‐world perspective, these findings raise important questions about how people currently engage with self‐management activities and the kinds of outcomes that can be expected from undertaking these activities. The timing of people's uptake of self‐management within the musculoskeletal disease continuum is an issue requiring further attention in both research and practice.

## Background

Self‐management has become a cornerstone to policy and practice development in the management of chronic diseases including muscu‐loskeletal (MSK) conditions.^1^ Within Australia, and in line with international trends, there is growing policy emphasis on self‐management as an essential component of chronic care models.^2^ Underpinning this drive to emphasize self‐management as part of a chronic condition treatment plan, is the assumption that with the correct ‘informational’ framing,^3^ an individual will be able to adopt and monitor choices to cope with and manage the progression of their condition^3^, ^4^ and thereby maintain their quality of life. As Greenhalgh *et al*.^3^ note, this ‘informational’ approach assumes that the provision of appropriate education will position every individual to be able to make rational and informed self‐management choices. It does not accommodate the processes involved in the embodied experience of living with a chronic condition or the wider social or cultural frames in which people experience and manage their health.^3^

Musculoskeletal conditions are highly prevalent, affecting 28% of the Australian population (over 6.5 million persons).^5^ They are wide ranging in aetiology and encompass acute (e.g. sprained ankle) and chronic conditions (e.g. arthritis).^5^ This heterogeneity is poorly understood at a community level,^6^ and impacts on the level of information accessed to self‐manage any MSK condition. This also makes it a challenge for effective management by health practitioners, especially as MSK conditions are often part of a multiple morbidity profile of patients.^7^, ^8^, ^9^ Despite the growing emphasis on self‐management as a key means of responding to the rising burden of MSK conditions at a population level, and its importance particularly within osteoarthritis (OA) clinical guidelines,^10^ there is a range of interpretations of what self‐management involves.^11^ This includes very formal activities such as participation in courses or programmes (commonly focused on education, physical activity and weight loss) to informal health practices initiated by the person to manage their condition within their particular context (i.e. the practical work of managing their condition, such as using an aid or going for a walk).^3^, ^12^

Uptake of formal self‐management programmes is limited^11^, ^13^ and the population reach of such programmes generally includes a greater proportion of women than men,^14^ those with socio‐economic advantage^8^ and higher education.^15^ There is a lack of consensus on the long‐term effectiveness of formal programmes: in part, this reflects the diversity of programmes offered, delivery modes utilized and evaluation methods. In the case of self‐management education (SME) programmes, a recent Cochrane review compared SME for OA with attention control or usual care.^16^ They found none to only small benefits using a range of outcome measures including pain, function and quality of life. Looking beyond formal programmes, it is difficult to assess the effectiveness and impact of independent self‐management activities in a community setting. Further, little is known about the profile of those who do not engage with self‐management activities. Indeed, there tends to be a presumption that if self‐management services and support are marketed in the correct way, all those eligible will participate and benefit.^2^, ^3^

The self‐management of MSK is largely focused on managing symptoms tied to the underlying pathological condition and limiting their impact on quality of life.^16^ However, quality of life measures have not been widely used to evaluate the effectiveness of self‐management for those with MSK conditions. This study seeks to provide a more detailed understanding of how people with chronic MSK conditions use a range of both formal and independent self‐management activities. It aims to assess the associations between the use of a comprehensive range of self‐management activities and quality of life using data obtained from a population health census and an associated computer‐assisted telephone interview (CATI) population survey targeting those with chronic MSK conditions.

## Methods

**Figure 1:**
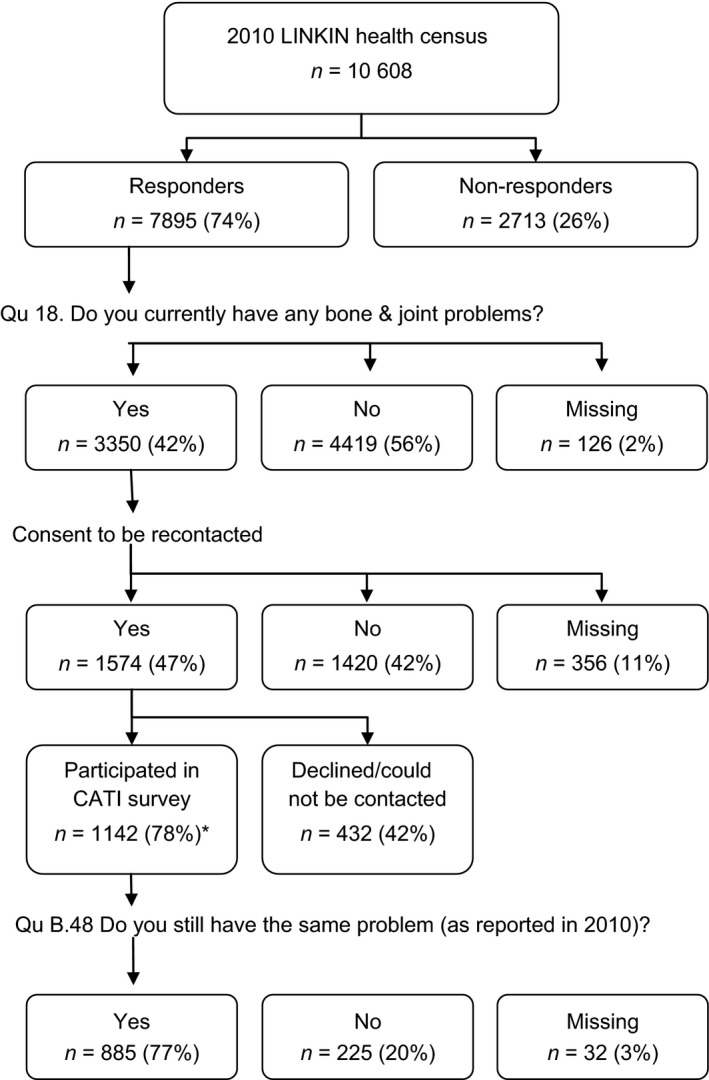
Flowchart of response rates. *Percentage of the eligible computer‐assisted telephone interview (CATI) sample that includes those health census respondents who consented to be recontacted minus those deceased or moved from the region.

The hypothesis being tested in this study is that people participating in self‐management activities for their chronic MSK condition are likely to have better quality of life than those who do not. In 2010, a health census of adults aged 15 years and over was conducted in Port Lincoln, a regional centre in South Australia (eligible population = 10 608; response rate 74%). The census methodology is described in detail elsewhere.^17^ Briefly, informed consent was gained via an information letter accompanying the questionnaires, which were hand delivered to all households. The letter advised potential respondents that participation was voluntary, that they had the right not to complete the questionnaire or any specific questions in the questionnaire and that returning the questionnaire would imply their informed consent. The census collectors delivering the questionnaires reinforced these messages to the household. The questionnaire collected data on socio‐demographic characteristics, current health conditions, health‐related quality of life (Short Form‐1 and EQ‐5D‐3L) and health service utilization, using a household and individual questionnaire for all residents aged 15 years and over. Subsequently, a CATI was conducted in 2012 with respondents who agreed to be recontacted (a census questionnaire question) and had reported a MSK condition in the census (*n* = 1142) (Figure 1). The interviews (which included verbal consent) obtained specific information about health service utilization, self‐management activities, and information seeking behaviour, in addition to demographic, quality of life and other health‐related information.^18^ The current study includes only the CATI respondents who reported still having the condition originally described in 2010 census (*n* = 885, with 10% of respondents being in the same household as one other respondent). Thus, the condition had now persisted for 18 months or more (i.e. was chronic). Although CATI interviews collected information about affected site(s) on the body, and whether a diagnosis had been given, this study does not exclude respondents without a formal diagnosis or restrict analysis to only certain MSK conditions. This inclusive selection criterion was chosen because it is important to understand how people manage the full range of chronic MSK conditions in a community setting where 11% had not visited a health provider about their condition (unpublished data).

### Measures

The exposure of interest was use of self‐management activities to manage their MSK condition (as assessed in the 2012 CATI). Respondents were asked five separate questions (with yes/no response options) as to whether they had used exercise, diet change, self‐management courses, community services/groups or other activities to manage the MSK condition reported in the 2010 health census. Where a response to ‘other self‐management activity’ was yes, the specific activity was described by the participant. As this question did not identify a cohesive group of self‐management activities, it was not analysed as a separate question but was included in the combined variable. Responses to these 5 separate questions were combined to create a dichotomous variable indicating whether they had undertaken at least one self‐management activity or none, to manage their MSK condition.

The primary outcome, health‐related quality of life, was measured using the new version of the EuroQOL five dimension questionnaire (EQ‐5D‐5L)^19^ which includes five levels of severity (no problems, slight problems, moderate problems, severe problems and extreme problems) in each of the five EQ‐5D dimensions (mobility, self‐care, usual activities, pain/discomfort, anxiety/depression).^20^ Data were collected for this outcome measure in 2012, as part of the CATI survey. Given that the self‐management of MSK conditions is largely focused on managing symptoms tied to the underlying pathological condition and their impact on quality of life,^16^ the EQ‐5D‐5L was chosen for this assessment.^21^ The EQ‐5D‐5L profiles were analysed using two methods. The first transformed the profiles, using the value set for the United Kingdom, into weighted health‐state index scores ranging from −0.594 (worst health‐state) to 1.00 (best health‐state).^22^ The second analyses tested for associations between self‐management activities and each of the dimensions separately.^23^ The levels of severity for each dimension were dichotomised into ‘no or slight problems’ and ‘moderate, severe or extreme problems’.

The analyses used variables that were identified, through bivariate analysis and existing literature,^1^, ^8^, ^14^, ^15^ as potentially influential of participation in self‐management activities. All independent variables that had a *P*‐value below 0.3 in the bivariate analysis or were identified from the literature as of potential importance (as in the case of comorbidities^24^) were included in the multivariable analysis. Co‐variables were categorized into five groups: socio‐demographic [sex, age, education level (year 10 or above vs. below year 10 which is equivalent to leaving school before 16 years of age)], marital status (married or de facto vs. living alone), smoking status (not a current smoker vs. current smoker), medical conditions (the presence of comorbidity) and self‐reported pain from the 2010 census data (no pain vs. at least moderate pain). These groups were used in the nested models described below. Ethics approval for the study was obtained from the University of Adelaide (H‐036‐2010).

### Data analyses

The proportion of respondents who undertook each of the nominated self‐management activities was determined. Descriptive analyses (using percentage and means, see Table 1) were undertaken to examine the characteristics of those who did not use, as well as those who did use specific self‐management activities. Regression methods were used to investigate the relationship between the use of at least one self‐management activity (combined variable) or specific types of self‐management activities, and health‐related quality of life, with cumulative adjustments for co‐variables. Stepwise forward selection of co‐variable groups was performed and their cumulative effects were examined. Nested generalized linear models with normal errors and identity link were used to test the association between health‐state index scores and specific self‐management activities, while adjusting for co‐variables. Tests for multicollinearity were conducted for all analyses, with variance inflation factors less than 2.5 for all models indicating low degrees of multicollinearity. Analyses were conducted for women and men separately if the participating proportions differed for a specific self‐management activity.^25^ Multivariable logistic regression using nested models tested the association of the dichotomized levels of severity for each of the five quality of life dimensions and those undertaking at least one self‐management activity, while adjusting for co‐variables. Regression coefficients (β) and 95% confidence intervals were used to evaluate the strength of the associations between self‐management activities and health‐state index scores. Odds ratios (OR) and 95% confidence intervals were used to evaluate the strength of the associations between self‐management activities and individual dimensions. All analyses were performed using stata, release 12.0 (Statacorp LP, College Station, TX, USA).

**Table 1:** Characteristics of total MSK populations (Census and CATI), including group profiles by whether they have used self‐management activities to manage their MSK conditions

	Total census population with MSK		Total CATI population with chronic MSK		Population who self‐manage		Population who do not self‐manage	
	*n*	%	*n*	%	*n*	%	*n*	%
Total	3350	100	885	100.0	666	76.1	210	24.0
Sex								
Male	1461	43.8	336	38.5	245	72.9	91	27.1
Female	1872	56.2	536	61.5	418	78.0	118	22.0
Marital status								
Married/de facto	2216	66.7	599	68.6	466	77.8	133	22.2
Not married/de facto	1109	33.4	274	31.4	198	72.3	76	27.7
Educational level								
Greater than Year 10	1765	54.9	476	55.5	372	78.2	104	21.9
Year 10 or less	1449	45.1	381	44.5	280	73.5	101	26.5
Smoking status								
Not a smoker	2560	78.5	721	83.9	549	76.1	172	23.9
Current smoker	700	21.5	138	16.1	104	75.4	34	24.6
Comorbidities								
No	1204	35.9	271	30.9	209	77.1	62	22.9
Yes	2146	64.1	605	69.1	457	75.5	148	24.5
EQ5D pain (2010)								
No/slight pain	713	21.7	152	17.6	107	70.4	45	29.6
Moderate or greater	2577	78.3	78.5	82.4	549	77.0	164	23.0

Missing data not included.

## Results

There were 885 respondents in the CATI survey, 61.5% were females, with a mean age of 58.4 years [standard deviation (SD) 14.9] [mean age of Census MSK population was 56.5 years (SD 17.7)]. Compared to the 2010 census MSK population (*n* = 3350), the CATI survey respondents were more likely to be female, non‐smokers, have comorbidities and report at least moderate pain (Table 1). With reference to participation in self‐management activities, 63% of CATI respondents reported having used exercise to manage their condition, 19% reported having used diet change, 12% a self‐management course, 3% community services/groups and 12% other approaches (e.g. rest and aids). Overall, 24% reported that they had not used any self‐management activities, and 66% stated that they had not sought any specific information about their chronic MSK condition (data not presented). Health‐state index scores ranged from −0.248 to 1 with a mean index score of 0.715. The descriptive statistics of the sample are presented in Tables 1 and 2.

**Table 2:** Health‐state index scores and age profiles associated with self‐management activities^a^

	Self‐management		Diet		Exercise		Exercise & diet		Formal support^c^	
	Yes	No^b^	Yes	No	Yes	No	Yes	No	Yes	No
Index score										
Mean	0.716	0.712	0.680	0.723	0.723	0.706	0.694	0.710	0.677	0.721
SD	0.193	0.224	0.208	0.198	0.186	0.223	0.210	0.223	0.222	0.198
Age										
Mean	57.3	62.0	55.2	59.1	57.2	60.4	54.7	60.7	56.7	58.7
SD	14.1	16.3	13.1	15.1	14.3	15.4	13.2	15.8	12.2	15.2

a Cases with missing data not included.

b No means did not use any of the following self‐management activities: diet, exercise, formal support (including self‐management course and community services/groups) and other activities.

c Formal support included use of self‐management course and/or community services/groups.

The first results presented compare quality of life index scores for respondents who reported using one or more of the listed self‐management activities, with respondents who had not used any of these activities. Results pertaining to different types of self‐management activities are then presented, to determine differences in quality of life between those who had used, and those who had not used specific self‐management activities.

### Use of self‐management activities

Quality of life did not differ greatly between people who did and did not undertake any of the self‐management activities. That is, self‐management activities showed no association with health‐state index scores, with and without adjustment for potential confounders (Table 3). The progressive adjustment for confounding factors (models 2–6) appeared to marginally increase the EQ‐5D‐5L index scores for the group not undertaking self‐management activities, but the final model which accounted for pain, attenuated this increase. As men were more likely than women to report lack of use of self‐management activities (Table 1), the use of self‐management activities was modelled separately by sex. There remained no association with health‐state index scores, with and without adjustment for confounders.

**Table 3:** Associations between use of self‐management activities and health‐state index scores for all and by sex

	All persons			Males			Females		
	ß	95% CI	*P*	ß	95% CI	*P*	ß	95% CI	*P*
Model 1	0.001	−0.035, 0.037	0.95	0.021	−0.030, 0.072	0.41	−0.017	−0.074, 0.022	0.51
Model 2	−0.013	−0.048, 0.022	0.46	−0.006	−0.046, 0.058	0.81	−0.026	−0.074, 0.022	0.29
Model 3	−0.016	−0.050, 0.019	0.38	−0.008	−0.044, 0.060	0.77	−0.031	−0.077, 0.015	0.19
Model 4	−0.017	−0.051, 0.017	0.32	0.006	−0.044, 0.057	0.80	−0.033	−0.078, 0.013	0.16
Model 5	−0.016	−0.050, 0.018	0.37	0.010	−0.041, 0.060	0.70	−0.032	−0.077, 0.014	0.17
Model 6	−0.005	−0.038, 0.029	0.78	0.014	−0.037, 0.064	0.59	−0.016	−0.061, 0.029	0.49

Model 1: Undertaking self‐management to manage MSK (0 = undertaking one or more activity, 1 = undertaking no activities). Model 2: Model 1 adjusted for age, education (year 10 or above vs. below year 10) and sex (male vs. female) for ‘all persons’ models. Model 3: Model 2 plus marital status (married or de facto vs. living alone). Model 4: Model 3 plus not current smoker vs. current smoker. Model 5: Model 4 plus comorbidity (does not have a comorbidity vs. has a comorbidity). Model 6: Model 5 plus self‐reported pain.

### Use of diet

People using dietary change activities to manage their MSK condition reported poorer quality of life relative to those not using dietary changes. That is, attempting diet change was negatively associated with health‐state index scores (Table 4). Progressive adjustment for confounders had little effect on the regression coefficient (models 2–6). When the individual dimensions of the EQ‐5D‐5L were analysed using logistic regression to examine associations between specific quality of life dimensions and diet changes, it was found that people using diet change activities were more likely to have at least moderate problems with walking about (OR: 1.87; 95% CI: 1.24–2.81; *P* = 0.003) and moderate problems with usual activities (OR: 1.63; 95% CI: 1.02–2.59; *P* = 0.040). Regression models run separately for men and women found that the negative associations between dietary change and index scores remained (Table 4).

**Table 4:** Associations between diet, exercise, and diet and exercise, with health‐state index scores

	All persons			Males			Females		
	ß	95% CI	*P*	ß	95% CI	*P*	ß	95% CI	*P*
Diet									
Model 1	−0.055	−0.091, − 0.019	0.003	−0.056	−0.112, −0.001	0.054	−0.058	−0.104, −0.012	0.014
Model 2	−0.064	−0.100, − 0.092	<0.001	−0.060	−0.116, −0.004	0.035	−0.067	−0.111, −0.022	0.004
Model 3	−0.064	−0.100, − 0.029	<0.001	−0.057	−0.112, −0.002	0.041	−0.068	−0.112, −0.023	0.003
Model 4	−0.064	−0.098, − 0.029	<0.001	−0.061	−0.115, −0.006	0.029	−0.062	−0.110, −0.017	0.007
Model 5	−0.062	−0.096, −0.028	<0.001	−0.061	−0.115, −0.008	0.025	−0.060	−0.105, −0.016	0.008
Model 6	−0.051	−0.085, − 0.017	0.003	−0.054	−0.107, −0.002	0.043	−0.047	−0.091, −0.004	0.034
Exercise									
Model 1	0.015	−0.014, 0.043	0.32	0.006	−0.037, 0.049	0.78	0.014	−0.025, 0.053	0.48
Model 2	0.004	−0.024, 0.032	0.78	0.006	−0.034, 0.045	0.79	0.003	−0.034, 0.041	0.86
Model 3	0.001	−0.027, 0.029	0.94	0.006	−0.037, 0.048	0.80	0.001	−0.038, 0.036	0.94
Model 4	0.002	−0.029, 0.025	0.88	0.004	−0.037, 0.047	0.83	0.007	−0.042, 0.029	0.71
Model 5	0.002	−0.029, 0.025	0.90	0.008	−0.034, 0.050	0.37	0.008	−0.044, 0.027	0.65
Model 6	0.003	−0.023, 0.030	0.80	0.010	−0.032, 0.051	0.66	0.000	−0.034, 0.034	0.99
Exercise and diet									
Model 1	−0.032	−0.077, − 0.013	0.17	−0.033	−0.105, 0.039	0.36	−0.034	−0.091, 0.023	0.25
Model 2	−0.048	−0.093, − 0.004	0.03	−0.041	−0.113, 0.031	0.27	−0.052	−0.107, 0.004	0.07
Model 3	−0.050	−0.095, − 0.006	0.03	−0.040	−0.119, 0.031	0.27	−0.057	−0.111, 0.003	0.04
Model 4	−0.060	−0.098, − 0.013	0.01	−0.056	−0.129, 0.016	0.13	−0.056	−0.110, −0.002	0.04
Model 5	−0.055	−0.097, − 0.013	0.01	−0.054	−0.125, 0.017	0.13	−0.056	−0.110, −0.003	0.04
Model 6	−0.042	−0.088, − 0.001	0.05	−0.048	−0.117, 0.022	0.18	−0.036	−0.089, 0.016	0.17

Model 1: Use of diet (or exercise, or diet and exercise) to manage MSK (e.g. 0 = not used diet, 1 = used diet). Model 2: Model 1 adjusted for age, education (year 10 or above vs below year 10) and sex (male vs. female) for ‘all persons’ models. Model 3: Model 2 plus marital status (married or de facto vs. living alone). Model 4: Model 3 plus not current smoker vs. current smoker. Model 5: Model 4 plus comorbidity (does not have a comorbidity vs. has a comorbidity). Model 6: Model 5 plus self‐reported pain.

### Use of exercise

Quality of life did not greatly differ between people who did and did not undertake exercise to manage their MSK condition. That is, the use of exercise showed no association with health‐state index scores, with and without adjustment for potential confounders, and these results were similar when the models were run separately for males and females (Table 4).

### Use of diet and exercise combined

As the most commonly reported combination of multiple self‐management activities, diet and exercise is of particular interest (14.8% reported this combination). People undertaking both dietary change and exercise to manage their MSK condition reported poorer quality of life relative to those who did not. When this combination was analysed, a negative association with health‐state index scores was observed (Table 4). Analysis of the individual EQ‐5D‐5L dimensions highlighted that people using this combination of self‐management activities were more likely to have at least moderate problems walking about (OR: 1.93; 95% CI: 1.06–3.50; *P* = 0.031), and moderate problems with usual activities (OR: 1.87; 95% CI: 1.03–3.41; *P* = 0.041). When models were run separately for males and females, similar negative associations were observed for each sex (Table 4).

### Use of formal support

People who used formal support to manage their MSK reported poorer quality of life relative to those who did not. That is, those who used formal support (a formal self‐management course and/or community services/groups) were more likely to have a negative association with health‐state index scores (Table 5). Progressive adjustment for confounders had little effect on the regression coefficient (models 2–6). Analysis of the individual EQ‐5D‐5L dimensions highlighted that people in this group were more likely to report at least moderate pain (OR: 1.58; 95% CI: 1.05–2.38; *P* = 0.028).

**Table 5:** Associations between formal support and health‐state index scores for all persons and by sex

Formal support^a^	All persons			Males			Females		
	ß	95% CI	*P*	ß	95% CI	*P*	ß	95% CI	*P*
Model 1	−0.052	−0.123, 0.015	0.15	−0.052	−0.123, 0.019	0.15	−0.036	−0.086, 0.014	0.16
Model 2	−0.049	−0.089, −0.009	0.02	−0.066	−0.137, 0.004	0.07	−0.040	−0.088, 0.009	0.11
Model 3	−0.050	−0.089, −0.010	0.01	−0.064	−0.133, 0.006	0.07	−0.042	−0.090, 0.006	0.09
Model 4	−0.048	−0.087, −0.009	0.02	−0.064	−0.133, 0.006	0.07	−0.038	−0.085, 0.008	0.11
Model 5	−0.044	−0.082, −0.005	0.03	−0.061	−0.129, 0.007	0.08	−0.033	−0.080, 0.014	0.17
Model 6	−0.040	−0.077, −0.002	0.04	−0.062	−0.128, 0.003	0.06	−0.027	−0.073, 0.019	0.25

Model 1: Used formal support to manage MSK (0 = not attended a self‐management course or community services/groups, 1 = has attended). Model 2: Model 1 adjusted for age, education (year 10 or above vs. below year 10) and sex (male vs. female) for ‘all persons’ models. Model 3: Model 2 plus marital status (married or de facto vs. living alone). Model 4: Model 3 plus not current smoker vs. current smoker. Model 5: Model 4 plus comorbidity (does not have a co‐morbidity vs. has a comorbidity). Model 6: Model 5 plus self‐reported pain.

a Formal support included use of self‐management course, and/or community services/groups.

## Discussion

Taking a whole population perspective, this study demonstrates that the most commonly reported self‐management activity with persisting MSK condition/s was exercise, followed by diet. There were low levels of reported participation in formal self‐management programmes, community services or group support activities. Of particular note, almost one‐quarter of respondents reported that they had not used any specific self‐management activities and almost two‐thirds had not accessed information to assist them in the management of their condition. It has been argued in other studies that for many people with arthritis, there is a belief that arthritis is something to be tolerated and not managed.^26^

A key finding of this study is that within this community setting, where independent self‐management activities (such as exercise and diet change) were more commonly used than formal support programmes, there is no association between the use of self‐management activities for MSK conditions, and quality of life. This finding raises important questions about how, in real‐world settings, people currently engage with activities; whether self‐management in a general community setting can be effective; and what should be expected from undertaking self‐management activities. These questions require more focused research including longitudinal studies of populations in community settings to establish casual relationships and pathways.

Given the growing policy emphasis on the provision of information and counselling about diet change and weight management for the management of arthritis^27^, ^28^ and other MSK conditions such as gout, the negative association between trying diet change and quality of life found in this study illuminates the challenges facing people undertaking these types of self‐management activities. Given that adjustments for multiple confounding factors did not change the associations and that problems with mobility and undertaking usual activities were important contributors to the negative association, this finding suggests that attempting diet change is generally not contributing to improved quality of life for these people with persisting MSK condition/s. Based on the results of the logistic regression, and following on from Hootman's observations that people with arthritis tend to seek out help only when things start to impact on valued life activities,^26^ it is suggested here that people may attempt diet change as their valued activities are affected by their condition, and their quality of life is in decline. However, it is recognized that sustained diet change and successful weight loss are difficult to achieve and sustain^29^ (especially beyond supported formal programmes), and therefore, any associated benefits in quality of life will be difficult to gain and retain. The cross‐sectional nature of this study means that it is not possible to identify causal pathways; more specific longitudinal studies would assist in unpacking the processes and pathways involved in diet change to manage MSK conditions and quality of life.

The negative association between use of formal support programmes and quality of life corroborates Kroon *et al*.'s^16^ contention that unlike other chronic conditions such as asthma and diabetes where poor management has clear demonstrable complications or deterioration in the conditions, OA (the most common of the MSK conditions) does not. This suggests that it is only when quality of life is substantially impacted that a ‘tipping point’ is reached and people seek help from formal support and programmes. Under such circumstances, it will be difficult to affect change in quality of life. These may be factors to consider in any redesign of chronic MSK care models.

This study has a number of important strengths, including the large population‐based data set, the range of variables covered and the administration of both surveys by trained personnel using a structured format. The cross‐sectional study design limits the analysis to associations: future population health studies which collect longitudinal data would be useful to examine issues of causality between MSK related self‐management activities and quality of life, including detailed measures of frequency, intensity and duration of self‐management activities. Study participants were potentially a heterogeneous group containing those who have a MSK lifelong diagnosis, such as OA, and those that have potentially resolvable MSK conditions. These groups may have responded differently to self‐management, but this study is unable to differentiate between them. Future research should consider potential differences in the response to self‐management between these groups. A further limitation of this study relates to the use of self‐reported data, including the respondents’ diagnoses of MSK conditions, and the potential response bias particularly in relation to those agreeing to be recontacted for the CATI survey. There may also be issues of generalizability given that the study site was a regional centre in Australia, particularly in relation to access to formal support and programmes.

## Conclusions

These findings do not sit comfortably with current policy discourse emphasizing the expectation that individuals undertake regular self‐management tasks to successfully manage chronic conditions to protect their quality of life. Given this current policy environment, the question becomes the following: What should we do to support people to manage their MSK condition? The inverse care law tells us that those with greatest need, as identified in this study, are less likely to act to address their care needs.^1^ Considering the burden of MSK conditions on the population, the argument remains that it is not sufficient or appropriate to ‘do nothing’ and assume that those who need support will access it. Self‐management as it is promoted currently may not be the only answer: People may benefit from a more proactive intermediary model to support and direct change in self‐care and effective use of resources earlier in the disease continuum. Thus, it may be necessary to rethink delivery of care to bridge the gap between one‐on‐one providers who are involved in early diagnosis and treatment, and a level of independence in the long‐term management of one's health that fits with people's personal priorities, preferences and values to support health‐related quality of life.

In conclusion, self‐management relies on the ability of people to access, understand and interpret a wide range of information. Use of self‐management activities will be influenced by a person's understanding of what works for them given the particular challenges and experiences of their social, economic and practical situation.^1^, ^30^ However, current approaches used by people with persisting MSK conditions in a community setting may be somewhat late in the disease continuum and not improve their quality of life.

## Funding

The LINKIN Health study is funded by an NHMRC Project grant 627240: The Physiology of Health Systems; Port Lincoln as a case study. Elizabeth Hoon is currently supported by Arthritis SA and the University of Adelaide's Florey Research Fund. Tiffany Gill is currently a National Health and Medical Research Council Early Career fellow.

## Conflict of interest

The authors declare that they have no competing interests.
